# Histological and Genetic Markers of Cellular Senescence in Keratinocyte Cancers and Actinic Keratosis: A Systematic Review

**DOI:** 10.3390/ijms27031520

**Published:** 2026-02-04

**Authors:** Piotr Sobolewski, Mateusz Koper, Anna Wasaznik-Jedras, Malgorzata Kolos, Irena Walecka

**Affiliations:** 1 Dermatology Clinic, National Medical Institute of the Ministry of Interior and Administration, 02-507 Warsaw, Poland; 2 Chair and Clinic of Dermatology and Pediatric Dermatology, Centre of Postgraduate Medical Education, 02-507 Warsaw, Poland; 3 Pathomorphological Center, National Institute of Medicine of the Ministry of Interior and Administration, 02-507 Warsaw, Poland

**Keywords:** cellular senescence, actinic keratosis, cutaneous squamous cell carcinoma, basal cell carcinoma, senescence biomarkers

## Abstract

Cellular senescence is a stress-induced cell-cycle arrest that constrains expansion of ultraviolet-damaged keratinocytes yet can remodel the microenvironment. This systematic review evaluated histological and genetic or epigenetic senescence markers in actinic keratosis (AK), cutaneous squamous cell carcinoma (cSCC), and basal cell carcinoma (BCC). PubMed, Scopus, and Web of Science were searched (January 2005–May 2025); 34 human studies were included. AK showed an early senescent signature with frequent cyclin-dependent kinase inhibitor p21 (p21CIP1) expression (82.1%) and DNA damage signaling, including phosphorylated histone H2AX (gamma-H2AX) positivity (77%). In invasive cSCC, p21^CIP1^ fell to 43.9% and tumor suppressor *p53* immunoreactivity often declined, whereas cyclin-dependent kinase inhibitor p16 (p16INK4a) commonly accumulated without arrest, including cytoplasmic staining at invasion fronts. Reported escape pathways involved c-Jun N-terminal kinase 2 activity and long noncoding RNA PVT1–dependent repression of p21. Telomerase reverse transcriptase (*TERT*) promoter mutations were prevalent in cSCC (about 50%) and BCC (up to 78%) but uncommon in AK, consistent with late telomerase activation. Study heterogeneity, variable antibody scoring, and limited assessment of senescence-associated beta-galactosidase and secretory mediators restricted cross-study comparability. Standardized, spatially resolved profiling may refine risk stratification and support senescence-targeted prevention and therapy in keratinocyte cancers.

## 1. Introduction

Cellular senescence is a permanent cell-cycle arrest program activated by stressors such as DNA damage, telomere attrition, or oncogene activation. This state of growth arrest is not merely a passive cessation of division but represents a metabolically active and complex physiological shift that serves as a fundamental tumor suppressor mechanism, particularly in the highly proliferative cells of the human skin. The initiation of this program involves a coordinated molecular cascade where the detection of irreparable genomic lesions triggers the activation of specialized signaling axes, most notably the ataxia telangiectasia mutated (*ATM*) and checkpoint kinase 2 (*Chk2*) pathways, which ultimately converge on the stabilization of *p53* and the transcriptional induction of cell cycle inhibitors [1]. Furthermore, the role of cellular senescence is increasingly recognized as a “double-edged sword” in the oncogenic landscape: while it prevents the immediate expansion of damaged clones, the long-term accumulation of these cells can foster a pro-tumorigenic microenvironment through the chronic release of diverse bioactive molecules [2]. Senescent cells upregulate cyclin-dependent kinase inhibitors (e.g., p16^INK4a^, p21^CIP1^) and secrete pro-inflammatory cytokines and proteases (the senescence-associated secretory phenotype, SASP). The SASP represents a distinct and heterogenous secretome that can vary significantly depending on the cell of origin and the nature of the senescent-inducing stimulus, potentially encompassing hundreds of proteins including interleukin-6 (IL-6), interleukin-8 (IL-8), and various matrix metalloproteinases (MMPs) that remodel the extracellular matrix [3]. Although the induction of senescence is often viewed as a terminal cell state, recent evidence suggests that certain aggressive tumor clones may develop sophisticated bypass mechanisms, utilizing signaling pathways such as the c-Jun N-terminal kinase 2 (JNK2) axis to suppress the p16-mediated barrier and maintain proliferative autonomy [2]. Although senescence limits proliferation of damaged cells, persistent senescent cells can disrupt tissue homeostasis and promote carcinogenesis through SASP factors. The implications of these secreted factors are particularly profound in the context of the tumor-stroma crosstalk, where senescent fibroblasts in the dermis can facilitate the expansion and migration of overlying malignant keratinocytes by providing a “pre-activated” niche rich in growth factors and inflammatory signals [4,5].

The skin is particularly vulnerable to UV-induced damage, and long-term UV exposure is the leading cause of photoaging and can lead to actinic keratosis and skin cancer [6]. The solar spectrum, specifically UVA (320–400 nm) and UVB (280–320 nm), exerts differential yet complementary roles in the induction of cutaneous senescence and malignant transformation [6]. UVA radiation is known to penetrate deeply into the dermal layers, where it generates reactive oxygen species (ROS) that induce oxidative stress and mitochondrial dysfunction, whereas UVB is primarily absorbed by the epidermis, causing direct DNA damage through the formation of cyclobutane-pyrimidine dimers [1]. The metabolic stress induced by these radiation types leads to a significant decrease in the expression of crucial nuclear fiber layer proteins, such as Lamin B1 (LMNB1), which is currently utilized as a sensitive and reliable biomarker for quantifying cellular senescence in photoexposed skin [6]. Modern therapeutic research is exploring the use of aloe vera gel and rind-derived nanoparticles (ADNPs) to mitigate these effects by activating the Nrf2/ARE pathway, which serves to alleviate oxidative stress and inhibit the typical elevation of bet-gal and SASP markers following UV insult [6]. UV exposure induces p16^INK4a^ expression in epidermal cells and drives senescence and SASP factor release [3]. In particular, repeated exposures to sub-cytotoxic levels of UVB have been shown to trigger a persistent DNA damage response (DDR) and the secretion of SASP factors that significantly accelerate the migratory capacity of cutaneous squamous cell carcinoma (cSCC) cells in both scratch and Boyden chamber assays [1].

Basal cell carcinoma (BCC) and cSCC are the most common non-melanoma skin cancers [7]. These malignancies are characterized by an extremely high mutation burden, with cSCC often exhibiting over 50 mutations per megabase of DNA, representing a massive expansion of simple mutation events demarcated by the recombinative loss of the second copy of *TP53* [8]. BCC, driven largely by Hedgehog-pathway mutations, is locally invasive but rarely metastatic. The slow growth kinetics and reduced metastatic potential of BCC, despite harboring many of the same genetic alterations as cSCC (such as *TERT* promoter mutations), suggest that lingering senescence programs or unique stromal interactions might limit its clinical aggressiveness [9]. cSCC is less common but more often fatal; it typically arises via a multistep process from precursor lesions such as actinic keratoses (AKs) [10]. The progression from AK to invasive cSCC involves the progressive subversion of the initial senescence brake, as cells acquire secondary hits in pathways such as *Notch* signaling or the MAPK cascade [8]. AKs are dysplastic keratinocyte proliferations (often visible as scaly patches) resulting from chronic UV exposure. Advanced molecular profiling, including single-molecule in situ hybridization (RNAscope), has demonstrated that precancerous lesions like AK already show a distinct nuclear localization of oncogenic long noncoding RNAs like PVT1, which acts as a suppressor of cellular senescence in the early stages of keratinocyte transformation [11]. Approximately 82% of cSCCs develop in fields of AKs, and AKs show early molecular alterations (e.g., *TP53* mutations) similar to invasive cSCC. The shared clonal origin between these lesions suggests that the “benign” behavior of AK is maintained by a functional senescence-associated secretory phenotype and checkpoint activation, which is ultimately lost during the transition to a fully malignant state [12].

In Gorlin syndrome (nevoid basal-cell carcinoma *syndrome*; NBCCS) patients with PTCH1 mutations, constitutive Hedgehog signaling makes even sun-protected skin prone to BCCs. In these patients, the underlying germline deficiency in PATCHED1 leads to a significant perturbation of epidermal differentiation and cell cycle control, even in the absence of external genotoxic stress. Stromal changes are also implicated: fibroblasts from Gorlin patients exhibit protumoral alterations that support BCC development. These patient-derived dermal fibroblasts secrete increased levels of sonic hedgehog (*SHH*) and matrix-degrading enzymes, creating a dermo-epidermal junction characterized by pathological clefting and delayed keratin expression [13]. Likewise, Gorlin patient-derived keratinocytes show enhanced Hedgehog signaling and resistance to UV-induced apoptosis, further predisposing to cancer [14]. The combination of these mesenchymal and epithelial defects suggests a co-evolutionary model of cancer development where the failure of normal senescence signaling in the stroma directly accelerates the transformation of the overlying tissue [4]. Stem cell regulators affect this balance: for instance, p63 is overexpressed in epidermis and promotes keratinocyte proliferation by inhibiting differentiation, apoptosis, and senescence [15,16]. This dominant isoform of the p63 family is essential for maintaining the proliferative capacity of basal keratinocytes, and its experimental downregulation using *small interfering RNA* (siRNA) has been shown to induce a significant G1-phase arrest accompanied by the expression of senescence-associated beta-galactosidase [11,17]. Understanding senescence markers in these lesions could inform diagnostics and risk stratification. Current research is increasingly focused on identifying functional elements within the genome, such as exon 2 of the PVT1 locus, which are critical for maintaining the oncogenic role of keratinocytes and preventing the onset of senescence [11]. Histological markers include nuclear accumulation of p16 or p21, H2AX foci (DNA damage), and senescence-associated beta-galactosidase (SA-beta-gal) activity. The localization of these markers within the tissue architecture is critical, as nuclear localization typically indicates active checkpoint signaling, whereas cytoplasmic accumulation—particularly of p16—may paradoxically indicate a failure of the senescence response [18]. Genetic/epigenetic markers include *TERT* promoter mutations, telomere shortening, SASP gene expression, and DNA damage signatures. The high frequency of *TERT* promoter mutations in both BCC (up to 78%) and cSCC (50%) underscores telomerase reactivation as a near-universal bypass of the replicative senescence mechanism triggered by telomere attrition [9]. Here we systematically review the evidence from human studies (2005–2025) on senescence-related markers in AK, cSCC, and BCC.

## 2. Methods

We adhered to PRISMA guidelines for systematic reviews. This structured approach ensures that the selection of evidence regarding the dynamic role of senescence in keratinocyte cancers is comprehensive and unbiased, allowing for a nuanced understanding of how diverse markers correlate with tumor stage [19].

Search strategy: We searched PubMed, Scopus, and Web of Science (January 2005–May 2025). The search string combined terms for senescence and keratinocyte lesions: e.g., ((“cellular senescence” OR “senescence-associated” OR “senescent”) AND (“actinic keratosis” OR “squamous cell carcinoma” OR “basal cell carcinoma” OR “keratinocyte carcinoma” OR “keratosis, actinic” OR “carcinoma, squamous cell” OR “carcinoma, basal cell”)). Boolean operators and synonyms were used, and Medical Subject Headings were applied where available. We limited results to English language and human studies. Reference lists of key articles were hand-searched for additional sources. (Supplementary Table S1).

Inclusion/exclusion criteria: We included original, peer-reviewed human studies that analyzed histological or genetic/epigenetic markers of cellular senescence in AK, cSCC, or BCC. Studies had to report marker data for human skin, dated 2005–2025, in English. We excluded: non-human studies, non-keratinocyte malignancies, case reports, case series, conference abstracts without data, and studies lacking relevant marker data. Studies focusing on unrelated skin conditions (e.g., melanoma) or outside skin were excluded. 

Screening process: Two reviewers independently screened titles and abstracts of all retrieved records. This dual-reviewer methodology helps mitigate selection bias and ensures the rigorous evaluation of papers. Discrepancies were resolved by discussion. Full texts of potentially eligible articles were then retrieved and assessed against inclusion criteria.

Data extraction: From each included study, two reviewers independently extracted data into a standardized form.

Data items included: author, year, country; study design (cohort, case series, case–control, etc.); patient population (number, demographics, sun-exposure status); lesion type (AK, BCC subtype, cSCC stage); methodology (histology/immunostains, molecular assays, sequencing techniques); markers analyzed; principal results (marker positivity rates, mutation frequencies, statistical comparisons). Any disagreements in extraction were resolved by consensus.

Quality assessment: The quality and risk of bias in included studies were evaluated independently by two reviewers. For genomic studies, we considered sample preparation, sequencing depth, and validation. We summarized quality as high, moderate, or low based on NOS scores. Any disagreements were resolved by discussion. 

**Figure 1:**
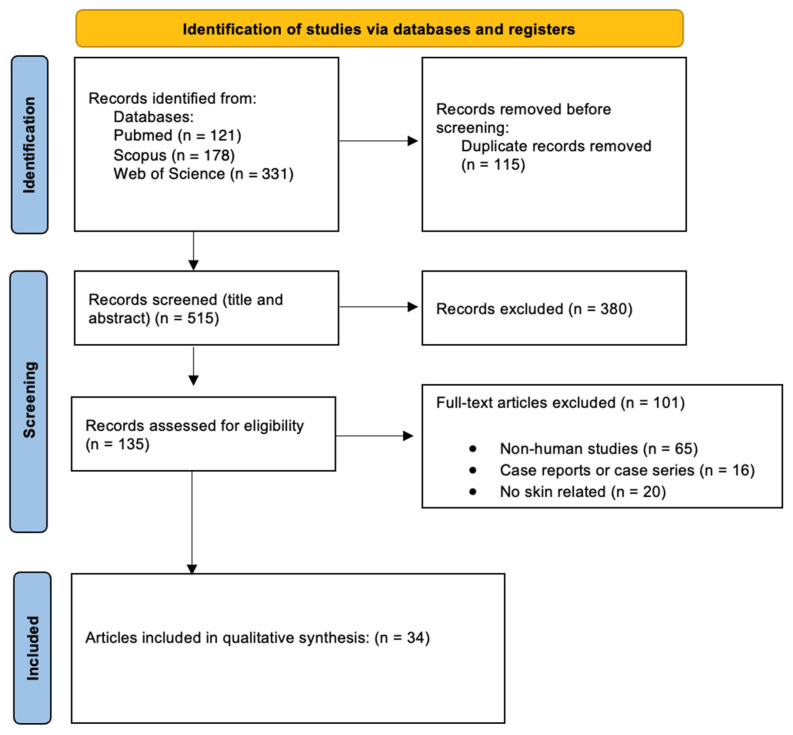
PRISMA flow diagram describing the selection of articles included in the review.

PRISMA flowchart: A PRISMA flow diagram (Figure 1) was generated to document the identification, screening, eligibility assessment, and final inclusion of studies in this review.

## 3. Results

The synthesis of the 34 included studies reveals a complex molecular map where senescence markers act as chronological indicators of keratinocyte transformation, beginning with the persistent DNA damage response found in photoaged skin and actinic keratosis [1] (Supplementary Table S2). These findings suggest that the metabolic roots of senescence—characterized by altered amino acid transport and cellular hypertrophy—are already established in pre-invasive lesions before the acquisition of major genetic hits like biallelic *TP53* loss [1,8]. Furthermore, the interaction between oncogenes like *MYC* and lncRNAs such as PVT1 creates a regulatory loop that prevents the induction of the senescence brake in developing squamous carcinomas [11].

### 3.1. Cell Cycle Inhibitor Markers (p16^INK4a^, p21^CIP1^, p53)

p16^INK4a^: Expression patterns in AK and cSCC were studied histologically. As a specific inhibitor of the cyclin D1-dependent kinases cdk4 and cdk6, p16 is normally absent in healthy stratified squamous epithelia but increases heterogeneously during the approach to the replicative limit [2,20]. AKs generally exhibited weak/cytoplasmic p16 staining with functional Rb (phosphorylated Rb), whereas squamous carcinoma in situ showed strong nuclear and cytoplasmic p16 with loss of Rb phosphorylation (nonfunctional Rb). In invasive cSCC, p16 expression was mixed: some tumors had strong cytoplasmic p16 and high proliferation, while invasive fronts showed upregulated cytoplasmic p16 independent of Rb status. This “senescence-marker positive” yet highly proliferative state is often driven by oncogenic JNK2, which acts to downregulate functional p16 levels and inhibit Ras-induced senescence programs [2]. Thus, p16 upregulation tended to occur at invasion fronts despite lack of growth arrest. In situ hybridization studies have confirmed that while p16 protein may accumulate, the mRNA transcripts are often dysregulated, and aggressive cSCC cells may acquire mutations that render them p16-insensitive [2]. Other studies noted increased p16 in oral and skin SCC as lesions became more dysplastic, but the proliferative marker Ki-67 often remained high, indicating p16 was not enforcing senescence [21].

No BCC-specific IHC studies of p16 met inclusion criteria. However, it is worth noting that BCC-associated fibroblasts exhibit a downregulation of *Notch* signaling effectors like CSL, which can induce a senescence phenotype in the stroma that paradoxically supports the growth of the tumor [4].

p21^CIP1^: As a direct downstream mediator of *p53*, p21 typically enforces arrest at the G1/S restriction point by inhibiting the activity of the cyclin-dependent kinase family [1,22]. Notably, 82.1% of AKs were p21-positive versus only 43.9% of invasive cSCCs, and high-intensity staining was seen only in well-differentiated or early-stage SCC. This high prevalence of p21 in AK supports its role as an essential part of the oncogene-induced senescence response that acts as an early barrier to malignancy [1]. This suggests p21-mediated arrest occurs early in carcinogenesis (AK and early SCC) and may be lost in later stages. The negative regulation of p21 by the lncRNA PVT1 is a significant mechanism for evasion; knockout of PVT1 or its specific exon 2 results in a dramatic upregulation of p21 and an accompanying increase in SA-beta-gal positive cells [11]. Other small studies have similarly found p21 expression in a majority of AKs and in situ cSCC, supporting its role as an early senescence marker in keratinocytes. Furthermore, p21 expression has been shown to be regulated by the insulin-like growth factor-1 receptor (IGF-1R), which is required for the proper induction of senescence following UVB radiation [1].

*p53*: *TP53* is the most frequently mutated gene in AK and cSCC. Temporal dissection of tumorigenesis reveals that decades of UV exposure and the inactivation of a single *TP53* allele result in a relatively stable epithelial exome, but the recombinative loss of the second allele triggers a vast expansion in mutagenesis [8]. UV-signature *TP53* mutations are often present even in histologically normal sun-exposed skin, and are found in nearly all AK and a high proportion of cSCC cases [23].

These ubiquitous C>T and CC>TT transitions represent a persistent “molecular scar” from solar radiation that disrupts the cell’s ability to trigger apoptosis or senescence [8]. Immunohistochemically, however, mutant *p53* protein tends to be more abundant in AK and precursor lesions than in invasive cSCC, which often lose *p53* staining due to truncating mutations. The physical interaction between CSL and *p53* in dermal fibroblasts illustrates how the loss of these tumor suppressors in the stroma can overcome fibroblast senescence and promote the expansion of malignant keratinocytes [4]. Indeed, one analysis found that cSCCs had slightly lower *p53* immunopositivity than adjacent AKs and markedly lower than normal epidermis. In advanced cSCC, secondary mutations in novel pathways, such as *Notch* and *PKHD1*, often follow biallelic *TP53* loss to drive malignant progression [8]. Overall, *TP53* pathway disruption is a nearly universal feature of AK to cSCC evolution. The persistent DDR signaling, characterized by increased phosphorylation of *p53* at Ser15, is maintained in senescent keratinocyte populations even 7 days after the final UV stress [1].

### 3.2. Other Senescence Markers

Gamma-H2AX (DNA damage marker): One study reported that 77% of AK specimens were immunopositive for gamma-H2AX, indicating DNA double-strand breaks, whereas seborrheic keratoses, Bowen’s disease (SCC in situ), BCC, and invasive cSCC showed little gamma-H2AX staining [24]. This DNA damage response is often associated with the overexpression of histone variant H2A.J, which accumulates in senescent cells and has been proposed to promote radioresistance and subsequent oncogenic transformation [10]. The high gamma-H2AX in AK supports the model that UV-induced DNA damage and senescence (oncogene-induced senescence) are prominent at precancer stages, whereas overt carcinomas may resolve these lesions through clonal selection. Persistently high levels of 53BP1 foci, which co-localize with various types of DNA damage, have also been observed in senescent keratinocytes, although they do not preferentially occur at telomeres in this specific cell type [1]. ADNPs have been validated in mouse models for their ability to alleviate this DNA damage through the activation of antioxidant defense mechanisms [6].

SA-beta-galactosidase: Surprisingly few studies applied SA-beta-gal assays to clinical keratinocyte lesions, likely due to technical limitations with fixed tissue. In laboratory settings, SA-beta-gal activity remains the “gold standard” for verifying senescence; for example, it was used to confirm that bitopertin-mediated inhibition of the *SLC6A9* transporter induces premature senescence in human keratinocytes [1]. As such, SA-beta-gal is not well characterized in human AK or cancer samples in the included literature. However, newer research utilizing freshly established xenograft models has demonstrated a significant increase in SA-beta-gal positive cells following the knockout of senescence-suppressing lncRNAs like PVT1 [11]. Similarly, in vivo photoaging studies in mice have successfully utilized SA-beta-gal to demonstrate the protective efficacy of plant-derived exosome-like nanoparticles [6].

Markers of mitochondrial dysfunction in keratinocytes: Mitochondrial dysfunction represents a central and increasingly recognized component of cellular senescence, particularly in keratinocytes exposed to chronic UV radiation [25]. UV-induced genotoxic stress triggers excessive production of reactive oxygen species (ROS), both directly through photochemical reactions and indirectly via mitochondrial electron transport chain (ETC) perturbation [26]. Keratinocyte mitochondria are especially vulnerable to oxidative damage due to their high metabolic activity and limited mitochondrial DNA (mtDNA) repair capacity, making mitochondrial impairment a key driver of senescence initiation and maintenance [27,28].

Experimental studies in human keratinocytes have demonstrated that repeated sub-cytotoxic UVB or UVA exposure leads to persistent mitochondrial ROS accumulation, loss of mitochondrial membrane potential (ΔΨm), and impaired oxidative phosphorylation [27,29]. These alterations promote a feed-forward loop in which mitochondrial dysfunction sustains the DNA damage response (DDR) and reinforces cell-cycle arrest via p53–p21^CIP1^ signaling [30]. Senescent keratinocytes exhibit reduced expression of mitochondrial biogenesis regulators, including peroxisome proliferator–activated receptor gamma coactivator 1-alpha (PGC-1α), along with decreased mitochondrial mass and altered mitochondrial dynamics characterized by increased fission and reduced fusion [31].

Several molecular markers have been proposed as indicators of mitochondrial dysfunction–associated senescence (MiDAS) in keratinocytes [32]. These include increased mitochondrial superoxide production, accumulation of mtDNA damage, and altered expression of mitochondrial quality control proteins such as PTEN-induced kinase 1 (PINK1) and Parkin [33,34]. UV-exposed senescent keratinocytes also display decreased levels of sirtuins (particularly SIRT3), which normally protect mitochondria from oxidative stress by regulating antioxidant enzymes such as superoxide dismutase 2 (SOD2) [35].

Although direct assessment of mitochondrial senescence markers in human actinic keratosis (AK), cutaneous squamous cell carcinoma (cSCC), and basal cell carcinoma (BCC) tissues remains limited, indirect evidence suggests their relevance. The high prevalence of persistent DDR signaling (e.g., γ-H2AX) and the senescence-associated secretory phenotype (SASP) in AK is consistent with mitochondrial ROS acting as a sustaining signal for senescence [36].

Recent therapeutic research further supports the role of mitochondrial dysfunction in cutaneous senescence. Activation of the Nrf2/ARE pathway, for example, by plant-derived nanoparticles, has been shown to reduce mitochondrial oxidative stress, restore mitochondrial homeostasis, and attenuate senescence-associated markers, including SA-β-gal activity and SASP components, in UV-damaged skin [37,38]. These findings indicate that mitochondrial health is a modifiable determinant of keratinocyte senescence and may influence the balance between tumor-suppressive and pro-tumorigenic outcomes.

### 3.3. Genetic and Epigenetic Markers

*TERT* promoter mutations: Studies consistently found frequent activating *TERT* promoter mutations in BCC and cSCC. Lead *TERT* mutations lead to increased expression of the catalytic subunit of telomerase, which stabilizes telomere length and genomic stability, effectively allowing cells to escape the replicative lifespan barrier [9]. Griewank et al. [8] reported *TERT* promoter mutations in 50% of invasive cSCCs and 78% of sporadic BCCs (68% in BCCs from nevoid basal cell syndrome). These mutations often show distinct UV-signatures (e.g., c.-124C>T), confirming that solar radiation is a causative factor in telomerase reactivation [9]. Pellegrini et al. similarly found *TERT* promoter mutations in 57.9% of BCCs [39]. The presence of these mutations in such a high percentage of non-melanoma skin cancers suggests that increased telomerase activity is a prerequisite for sustained tumor growth and avoidance of telomere-crisis-induced apoptosis [9]. In AK, these mutations were much rarer (1/11 cases of Bowen’s disease in Griewank et al. [8]), suggesting telomerase reactivation is a late event or marks progression beyond AK. Longitudinal single-cell analyses have shown that *TERT* mutations can occasionally occur in early AK clones, suggesting they may designate a subset of lesions with a higher risk of malignant transformation [12].

Telomere length/attrition: None of the included studies directly measured telomere length in AK or keratinocyte cancers. However, Q-FISH analysis of human skin has revealed that telomeres in the epidermis shorten at an annual rate of approximately 36 base pairs, and this process is significantly accelerated by sun exposure. Interestingly, telomeres in the basal layer are not always the longest, suggesting that epidermal stem cells may not be exclusively restricted to the basement membrane niche [40]. The high *TERT* mutation rates imply that bypass of replicative senescence via telomerase upregulation is common in these tumors. In advanced SCC, telomere dysfunction constraint is often bypassed through the inactivation of checkpoint genes, allowing genomic instability to fuel the most aggressive stages of tumor evolution [9].

SASP and inflammatory markers: No included human study systematically profiled SASP factors in situ. Nevertheless, research on conditioned media has shown that UVB-induced senescent keratinocytes secrete a protumoral SASP rich in IL-6 and IL-8, which significantly enhances the migratory potential of adjacent SCC cells [1,41]. A few reports noted increased local inflammation in AK fields, but specific cytokine or MMP markers of senescence were not quantified in tumor samples. In vivo, the protective effect of ADNPs is partially mediated through the inhibition of these inflammatory SASP components, highlighting them as promising therapeutic targets for preventing photoaging and subsequent carcinogenesis [6]. Furthermore, the downregulation of amino acid transporters has been identified as a novel transcriptomic hallmark of the senescent state, potentially influencing the metabolic profile of the SASP [1].

RF4, TYR, and DEF8 genes: Genes such as *IRF4* (likely corresponding to RF4), *TYR*, and *DEF8* have emerged as key genetic risk factors for actinic keratosis (AK) through large-scale genome-wide association studies (GWAS) in populations of European ancestry [42].

The *IRF4* variant rs12203592, located on chromosome 6p25, shows the strongest association with AK severity, influencing enhancer-mediated transcriptional regulation and pleiotropically affecting pigmentation and oncogenic pathways independent of skin color. Similarly, the *TYR* variant rs1126809 on chromosome 11q14 increases AK risk, modulating melanin synthesis via tyrosinase activity and correlating with reduced tanning response [43].

At the 16q24 locus, *DEF8* and the independent signal SPATA33 represent novel susceptibility signals, distinct from *MC1R*, implicated in pigmentation, tanning, and progression to cutaneous squamous cell carcinoma (cSCC) [43].

These loci, enriched in melanin biosynthesis pathways, explain part of AK’s heritability and highlight pigmentation-immune interactions as central to UV-driven keratinocyte neoplasia, supporting their utility as biomarkers for risk stratification.

Other mutations and clonal markers: A 2025 single-cell sequencing study (Nassir et al. [11]) found that early AKs harbored recurrent *CDKN2A* (p16) and *TERT* promoter mutations not seen in normal skin, while progression to cSCC involved acquisition of additional hits (e.g., *ARID2* and MAPK pathway genes). The identification of recurrent biallelic inactivation in tumor suppressors like *NOTCH1* and *NOTCH2* underscores how expanded mutagenesis facilitates the evasion of multiple senescence and differentiation checkpoints [8]. This implies that loss of senescence regulators (p16) and telomere reactivation are key early steps. Furthermore, the c-Jun N-terminal kinase (JNK) family has been shown to play a dominant role in human epidermal neoplasia, with JNK2 suppressing p16 and NF-kappa-B activation to transform primary cells into malignancy [2,44]. Notably, the study also observed that AKs often arose from the same progenitor clones as cSCC, underscoring clonal evolution. The antagonistic roles of JunB and c-Jun in Ras-driven neoplasia further illustrate how the balance between proliferation and senescence is meticulously tuned by the AP-1 transcription factor family [2,45].

Epigenetic alterations: Very few studies examined DNA methylation or chromatin marks related to senescence in these lesions. Long noncoding RNAs have emerged as essential regulators of the epigenetic landscape in SCC; for example, PVT1 transcription is regulated by *MYC* and its expression level correlates with the malignant phenotype [11]. One genome-wide association study identified risk alleles (e.g., near *IRF4*, *MC1R*) for AK/cSCC but did not directly address senescence pathways. The discovery that miR-124 acts as a tumor-suppressive miRNA in cSCC and is transcriptionally regulated by activated MAPK signaling provides a clear link between oncogenic drivers and epigenetic suppression of transformation [46]. This remains an area for future research.

### 3.4. Comparisons Between AK and Keratinocyte Cancers

Across studies, AKs appeared to harbor early senescence-related changes: they often had mutated *TP53*, frequently expressed p21, and high gamma-H2AX, indicating active DNA damage responses and temporary arrest. This “senescent” profile in AK is consistent with a population of cells that has undergone oncogene-induced senescence in response to chronic UV radiation but has not yet acquired the secondary mutations necessary to bypass the replicative limit [1]. In contrast, invasive cSCCs demonstrated loss of some of these markers (reduced p21, lower *p53* immunostaining) and acquired proliferative features. For example, Natarajan et al. observed that strong p16 expression was uniform in carcinoma in situ but variable in invasive SCC, and was often cytoplasmic at invasion fronts, suggesting cancer cells may evade senescence despite expressing p16 [47]. Transcriptomic comparisons reveal that senescent keratinocytes share more expressed genes with AK samples than with metastatic cSCC, reinforcing the temporal sequence of biomarker loss [1,12,48]. BCCs differed further: they less frequently studied senescence markers histologically, but genomically they exhibited high rates of mutation (similar to cSCC). Activating *TERT* mutations in BCC allow these slow-growing tumors to maintain genomic stability over decades without progressing to an aggressive metastatic phenotype [9]. BCCs also had intermediate *TP53* mutation frequency (~46%). The specific protein expression profiles of BCC, which include reduced collagen XVII and pan-desmoglein levels, correlate with its unique *de novo* dermal invasion pattern compared to the progressive invasion seen in SCC development [19]. In summary, AKs show early oncogene-induced senescence signals (*p53* response, p21), whereas cSCCs and BCCs accumulate mutations (*TP53*, *TERT*) that ultimately bypass or disable these checkpoints.

### 3.5. Methodological Variability

The included studies varied widely. Histological marker studies typically used immunohistochemistry (IHC) on formalin-fixed biopsies and scored staining semi-quantitatively (e.g., H-score). The use of different antibody specifications, such as clone 6H12 for p16INK4a or clone SX118 for p21WAF1, often limits the direct comparison of H-scores between independent cohorts [19]. Antibody clones and thresholds differed between studies, limiting direct comparisons. High-impact research has successfully integrated SNP array data and exome sequencing to reconstruct the genomic history of individual tumors, providing a more reliable timeline than mutation-by-stage approaches [8]. Several studies lacked matched normal skin controls, which limits interpretation of “overexpression.” DNA studies ranged from targeted PCR/sequencing (for *TERT*, *TP53* hotspots) to whole-exome or genome sequencing. Advanced bioinformatics tools like DESeq2 and Gene Set Enrichment Analysis (GSEA) have allowed for the identification of hundreds of differentially expressed genes in senescent keratinocyte populations [1,11]. Coverage and sensitivity varied; some single-cell approaches achieved high depth but limited samples. Strengths across studies included the use of human clinical specimens and, in some cases, quantification by multiple observers. Research on plant-derived nanoparticles has introduced innovative in vivo tracking methods, such as small animal live imaging of fluorescently labeled ADNPs, to validate their skin permeability [6]. Limitations included small sample sizes (<20 lesions in many reports), cross-sectional design (few longitudinal or functional validations), and heterogeneous patient populations (age, UV exposure). Quality assessment rated most studies as moderate: they often clearly defined assays and outcomes, but lacked adjustments for confounders (e.g., patient age or lesion location) and sometimes had incomplete reporting of negative findings. Future investigations using standardized Tissue Microarrays will be essential for validating these histological markers in larger, outcome-matched cohorts [19].

## 4. Discussion

This systematic review synthesized evidence on senescence markers in keratinocyte carcinogenesis. A coherent picture emerges of dynamic changes in cell cycle regulators and DNA damage responses across progression from sun-exposed skin to AK to carcinoma. Senescence is currently viewed as a two-stage mechanism in keratinocytes: an initial p16-dependent arrest followed by a *p53*-dependent enforcement that is independent of telomere status [9].

### 4.1. Histological Markers

p21^CIP1^, a *p53* target, was frequently positive in AK and early SCC but diminished in advanced tumors. This supports the paradigm of oncogene-induced senescence (OIS) acting as an early brake that is later subverted. The inhibition of amino acid transporters like *SLC6A9* has been shown to prematurely induce these p21-mediated traits, suggesting that metabolic regulation is a key tuning mechanism for senescence [1]. In contrast, p16^INK4a^ expression paradoxically increased in many progressing lesions. Azazmeh et al. found p16 strongly upregulated in carcinoma in situ, but notably tumor cells at the invasive front accumulated cytoplasmic p16 without arrest [18]. This p16-positive but non-arrested phenotype may be driven by oncogenic signaling from the JNK2 pathway, which suppresses the normal senescence-inducing function of p16 and permits continued proliferation [2]. Thus, although p16 is a canonical senescence marker, cancer cells may mislocalize it and override its effects. The coordination of p16 expression with Laminin 5 at the migrating fronts of wounds and early invasive lesions suggests that this coupling is a normal physiological response that goes awry during neoplastic progression [47]. This “senescence marker-positive” phenotype is recognized in other cancers as indicating aggressive clones that escaped arrest. *TP53* protein (often mutant *p53*) was abundant in AK but often lost or dysfunctional in invasive cSCC, reflecting clonal selection for cells bypassing the p53 checkpoint. The discovery that vast mutational expansion in cSCC is sharply demarcated by the loss of the second *TP53* copy reframes the *p53* protein as a critical “gatekeeper” of genomic stability [8]. Gamma-H2AX staining confirmed extensive DNA damage in AK, consistent with UV-induced OIS. The accumulation of Histone variant H2A.J in senescent populations further contributes to a radioresistant phenotype that may complicate the treATMent of advanced keratinocyte cancers [10]. Surprisingly, SA-beta-gal assays were rarely applied, highlighting a gap: future studies could adapt senescence-associated staining to fresh/frozen skin samples. Recent animal research has demonstrated that ADNPs can effectively reduce the percentage of SA-beta-gal positive cells in irradiated skin by activating the Nrf2 antioxidant pathway, offering a potential strategy for anti-senescence therapy [6].

### 4.2. Genetic and Epigenetic Markers

The high prevalence of *TERT* promoter mutations in BCC and cSCC underscores telomerase reactivation as a near-universal escape from replicative senescence [49]. Activating *TERT* mutations maintain telomere length and allow the continuous division of malignant clones, providing a mechanism for long-term survival and invasiveness [9]. That *TERT* mutations were much less common in AK suggests that bypass of replicative senescence occurs late, likely during transition to invasive carcinoma. Detailed Q-FISH measurements indicate that AK lesions arise from epidermis with excessively shortened telomeres, suggesting that telomere shortening itself is a primary driver of the initial precancerous transformation [40]. Single-cell analyses revealed that loss-of-function *CDKN2A* (p16) often co-occurs with *TERT* mutation in AK, implying these changes enable clonal expansion of dysplastic cells. Biallelic inactivation of *PKHD1* and secondary mutations in novel pathways further contribute to the aggressive mutational exome of cSCC [8]. In cSCC, additional oncogenic pathways (e.g., RAS/MAPK) are acquired. The deregulation of miR-130a by the MAPK pathway illustrates an epigenetic layer where tumor suppressive miRNAs are silenced to facilitate keratinocyte transformation [46,50]. *TP53* mutations, ubiquitous in AK and SCC, presumably precede or accompany senescence bypass. The finding that Decades of UV damage and a single *TP53* hit only result in about one hundred mutations underscores the tenacious genetic stability of human skin before the final checkpoint breakdown [8]. Few studies addressed epigenetic marks; given the role of epigenetics in regulating senescence (e.g., heterochromatin in SAHF), this is a major gap. However, the role of long noncoding RNAs like PVT1 in regulating *MYC* targets and suppressing p21 demonstrates that lncRNAs are critical players in the epigenetic landscape of cSCC [11].

### 4.3. AK vs. Carcinoma

Comparatively, AKs exhibited a “senescent” signature (high DNA damage, checkpoint activation) that is gradually lost as tumors evolve. The transition from AK to cSCC is characterized by a failure of the tumor-suppressive *p53*-p21 axis, as malignant clones selectively outcompete cells that remain capable of arrest [1]. The drop in p21 and *p53* staining from AK to invasive cSCC suggests that malignant cells selectively eliminate cells capable of arrest. Furthermore, the downregulation of amino acid transporters in senescent cells suggests that metabolic exhaustion may contribute to the eventual breakdown of the senescence response in precancerous fields [1,51]. In BCC, which may arise from basal epidermis without an obvious precursor field, senescence markers are less studied histologically. Studies of NBCCS fibroblasts suggest that mesenchymal-ectodermal interactions and a “pre-activated” stromal state are primary drivers of BCC development, often independent of direct UV-induced mutation [13]. However, molecularly BCCs share key features: frequent UV signatures (e.g., *TP53*) and *TERT* mutations, indicating they too bypass telomere- and DNA damage–induced checkpoints. Importantly, unlike cSCC, BCC rarely metastasizes despite these changes, suggesting senescence programs may limit its aggressiveness in ways yet to be understood. The interaction between CSL and *p53* in the stroma highlights how the loss of convergent control can lead to CAF activation and the expansion of keratinocyte-derived tumors [4] (Table 1).

### 4.4. Methodological Critique

The evidence base has strengths (use of real patient tissues, inclusion of multiple lesion types) but also limitations. One major limitation is the reliance on cross-sectional analysis, which may not capture the dynamic flux of biomarkers like SASP factors during the lifelong evolution of a tumor [19]. IHC studies often had no objective quantitation, and differences in antibody specificity and scoring make cross-study comparisons difficult. Standardized IHC scoring criteria—such as those based on the intensity and percentage of p16-positive cells—must be adopted to ensure the comparability of future studies [19]. Many genetic studies sequenced limited gene panels, potentially missing novel senescence regulators. The application of single-molecule in situ hybridization (RNAscope) has recently provided more precise quantification of biomarkers like PVT1 in paraffin-embedded tissue, overcoming some of the traditional limitations of histological assessment [11]. There was also a focus on advanced or symptomatic lesions; early or regressing AKs are understudied. The development of plant-derived nanoparticles and their evaluation in both in vitro and in vivo models provides a new framework for investigating the pharmacological mitigation of the photoaging process [6]. Most data are cross-sectional—longitudinal studies would better capture the temporal sequence of marker changes. Sample sizes were typically small, raising risk of false-negative findings. Future multi-center studies utilizing deep sequencing and spatial transcriptomics will be necessary to fully map the senescent cell populations within the complex cutaneous microenvironment [8,12].

Markers include cell-cycle regulators, DNA damage indicators, and genomic alterations implicated in senescence induction or escape. Expression patterns are based on immunohistochemical and molecular findings from human studies published between 2005 and 2025. Abbreviations: AK, actinic keratosis; cSCC, cutaneous squamous cell carcinoma; BCC, basal cell carcinoma; IHC, immunohistochemistry; SASP, senescence-associated secretory phenotype.

Most histological markers were assessed using immunohistochemistry on formalin-fixed, paraffin-embedded (FFPE) skin biopsies, whereas genetic alterations were detected using targeted or next-generation sequencing approaches (Table 2). The absence of methods for certain markers reflects gaps in the current literature rather than biological absence.

## 5. Conclusions

Cellular senescence serves as a pivotal checkpoint in the lifelong struggle against cutaneous carcinogenesis, initially acting as a defensive barrier in actinic keratosis before being systematically dismantled during the progression to invasive squamous cell carcinoma. The transition from the high-p21 “senescent signature” found in pre-invasive lesions to the biallelic-*TP53* loss and telomerase reactivation observed in malignant tumors designates a clear molecular timeline for keratinocyte transformation. Key histological indicators such as nuclear p16 and genetic events like *TERT* promoter mutations are currently the most reliable tools for identifying aggressive clones that have acquired replicative immortality. Furthermore, the role of the cutaneous stroma—particularly the activation of cancer-associated fibroblasts through the loss of *p53* and *Notch* signaling—highlights that the tumor microenvironment is as critical to cancer progression as the mutations within the keratinocytes themselves. Recent advances in nanomedicine, specifically the activation of the Nrf2/ARE pathway by aloe-derived nanoparticles, offer a promising preventative avenue for delaying photoaging and the accumulation of senescent cells. Future research should prioritize longitudinal monitoring and single-cell spatial profiling to distinguish “benign” senescence from the pro-tumorigenic SASP state, ultimately facilitating the development of precise senolytic and senomorphic therapies for the management of keratinocyte cancers.

## Figures and Tables

**Table 1:** Senescence-associated markers in AK, cSCC, and BCC.

Marker	Biological Level Assessed	Detection Method(s)	Sample Type
**p16INK4a (CDKN2A)**	Protein (expression, localization)	Immunohistochemistry (IHC)	FFPE skin biopsies
**p21CIP1 (*CDKN1A*)**	Protein	Immunohistochemistry (IHC)	FFPE skin biopsies
***p53* (*TP53* protein)**	Protein accumulation	Immunohistochemistry (IHC)	FFPE skin biopsies
***TP53* mutations**	DNA sequence	Targeted PCR and sequencing	FFPE or fresh-frozen tissue
**γ-H2AX**	Protein (DNA damage foci)	Immunohistochemistry (IHC)	FFPE skin biopsies
**SA-β-galactosidase**	Enzymatic activity	Histochemical staining	Fresh or frozen tissue
***TERT* promoter mutations**	DNA sequence	PCR with Sanger or NGS sequencing	FFPE or fresh-frozen tissue
**Telomere length**	Chromosomal structure	Not assessed in included studies	—
**SASP factors**	Protein/transcript	Not systematically assessed	—
**CDKN2A loss**	Genomic alteration	Single-cell or targeted sequencing	Fresh tissue

**Table 2:** Methods used for detection of senescence-associated markers in AK, cSCC, and BCC.

Marker	Type of Marker	Actinic Keratosis (AK)	Cutaneous Squamous Cell Carcinoma (cSCC)	Basal Cell Carcinoma (BCC)	Interpretation in Disease Progression
**p16^INK4a^**	Cell-cycle inhibitor (CDKN2A)	Weak or cytoplasmic expression; often functional Rb	Variable; strong in SCC in situ, cytoplasmic at invasive fronts	Limited histological data	
**p21^CIP1^**	Cell-cycle inhibitor (*CDKN1A*)	Frequently positive; high in early lesions	Reduced in invasive tumors	Not well characterized	Early DNA damage–induced senescence
***p53*** **/*TP53***	Tumor suppressor; DNA damage response	Most frequently mutated gene in AK	Mutated in most cases; reduced IHC in invasive SCC	Intermediate mutation frequency	Early checkpoint loss in progression
**γ-H2AX**	DNA damage marker	Frequently positive	Low or absent	Low or absent	UV-induced DNA damage in precancerous lesions
***TERT* promoter mutations**	Telomerase reactivation	Rare	Frequent	Very frequent	
**SASP factors**	Pro-inflammatory secretory phenotype	Suggested but not quantified	Not systematically assessed	Not assessed	

## Data Availability

No new data were created or analyzed in this study. Data sharing is not applicable to this article.

## Supplementary Materials

The following supporting information can be downloaded at: https://www.mdpi.com/article/10.3390/ijms27031520/s1.
