# Supplementary material for: Histological and Genetic Markers of Cellular Senescence in Keratinocyte Cancers and Actinic Keratosis: A Systematic Review
Source: Int J Mol Sci. 2026 Feb 4;27(3):1520. doi: 10.3390/ijms27031520 (PMC12898786; doi:10.3390/ijms27031520)
Supplement: Supplementary file 1 [file ijms-27-01520-s001.zip › Supplementary Table S1.pdf]

**Supplementary Table S1.** Search strategy

For Pubmed:
("cellular senescence"[MeSH Terms] OR senescen*[tiab] OR "senescent cell*"[tiab] OR "replicative senescence"[tiab] OR "cellular aging"[tiab])
AND
(marker*[tiab] OR biomarker*[tiab] OR "molecular marker*"[tiab] OR "genetic marker*"[tiab] OR "histological marker*"[tiab])
AND
(p16[tiab] OR p16INK4a[tiab] OR CDKN2A[tiab] OR p53[tiab] OR TP53[tiab] OR p21[tiab] OR CDKN1A[tiab] OR "SA-beta-gal"[tiab] OR "senescence-associated beta galactosidase"[tiab] OR "beta-galactosidase"[tiab] OR telomere*[tiab] OR telomerase[tiab] OR TERT[tiab] OR SASP[tiab] OR "senescence-associated secretory phenotype"[tiab] OR lipofuscin[tiab] OR "lamin B1"[tiab] OR LMNB1[tiab] OR HMGB1[tiab] OR "gamma-H2AX"[tiab] OR "γH2AX"[tiab])
AND
(("Carcinoma, Basal Cell"[MeSH Terms] OR "basal cell carcinoma"[tiab] OR BCC[tiab] OR basalioma[tiab]) OR ("Carcinoma, Squamous Cell"[MeSH Terms] OR "squamous cell carcinoma"[tiab] OR SCC[tiab] OR cSCC[tiab] OR "cutaneous squamous cell carcinoma"[tiab] OR "epidermoid carcinoma"[tiab]) OR ("Keratosis, Actinic"[MeSH Terms] OR "actinic keratosis"[tiab] OR "solar keratosis"[tiab] OR AK[tiab]) OR ("nonmelanoma skin cancer"[tiab] OR "non-melanoma skin cancer"[tiab] OR NMSC[tiab] OR "keratinocyte carcinoma"[tiab]))
AND english[lang]
AND Humans[MeSH Terms]

For Web of science:
TS=((senescen* OR "cellular senescence" OR "senescent cell*" OR "replicative senescence" OR "cellular aging") AND (marker* OR biomarker* OR "molecular marker*" OR "genetic marker*" OR "histological marker*") AND (p16 OR p16INK4a OR CDKN2A OR p53 OR TP53 OR p21 OR CDKN1A OR "SA-beta-gal" OR "beta-galactosidase" OR telomere* OR telomerase OR TERT OR SASP OR "senescence-associated secretory phenotype" OR lipofuscin OR "lamin B1" OR LMNB1 OR HMGB1 OR "gamma-H2AX" OR γH2AX) AND ("basal cell carcinoma" OR BCC OR "squamous cell carcinoma" OR SCC OR cSCC OR "actinic keratosis" OR AK OR "solar keratosis" OR NMSC OR "non-melanoma skin cancer" OR "keratinocyte carcinoma"))
Refine: Languages = English

For Scopus:
TITLE-ABS-KEY((senescen* OR "cellular senescence" OR "senescent cell*" OR "replicative senescence" OR "cellular aging")
AND (marker* OR biomarker* OR "molecular marker*" OR "genetic marker*" OR "histological marker*")
AND (p16 OR p16INK4a OR CDKN2A OR p53 OR TP53 OR p21 OR CDKN1A OR "SA-beta-gal" OR "beta-galactosidase" OR telomere* OR telomerase OR TERT OR SASP OR "senescence-associated secretory phenotype" OR lipofuscin OR "lamin B1" OR LMNB1 OR HMGB1 OR "gamma-H2AX" OR "γH2AX")
AND ("basal cell carcinoma" OR BCC OR "squamous cell carcinoma" OR SCC OR cSCC OR "actinic keratosis" OR AK OR "solar keratosis" OR NMSC OR "non-melanoma skin

cancer" OR "keratinocyte carcinoma"))
AND (LIMIT-TO(LANGUAGE,"English"))
