# Supplementary material for: Histological and Genetic Markers of Cellular Senescence in Keratinocyte Cancers and Actinic Keratosis: A Systematic Review
Source: Int J Mol Sci. 2026 Feb 4;27(3):1520. doi: 10.3390/ijms27031520 (PMC12898786; doi:10.3390/ijms27031520)
Supplement: Supplementary file 1 [file ijms-27-01520-s001.zip › Supplementary Table S2.pdf]

Supplementary Table S2. Publications included.

1: Sun Z, Zheng Y, Wang T, Zhang J, Li J, Wu Z, Zhang F, Gao T, Yu L, Xu X, Qian H, Tan Y. <i>Aloe Vera</i> Gel and Rind-Derived Nanoparticles Mitigate Skin Photoaging via Activation of Nrf2/ARE Pathway. Int J Nanomedicine. 2025 Apr 2;20:4051-4067. doi: 10.2147/IJN.S510352. PMID: 40191040; PMCID: PMC11972608.

2: Ventura A, Pellegrini C, Cardelli L, Rocco T, Ciciarelli V, Peris K, Fargnoli MC. Telomeres and Telomerase in Cutaneous Squamous Cell Carcinoma. Int J Mol Sci. 2019 Mar 16;20(6):1333. doi: 10.3390/ijms20061333. PMID: 30884806; PMCID: PMC6470499.

3: Todorova K, Mandinova A. Novel approaches for managing aged skin and nonmelanoma skin cancer. Adv Drug Deliv Rev. 2020 Jan 1;153:18-27. doi: 10.1016/j.addr.2020.06.004. Epub 2020 Jun 8. PMID: 32526451.

4: Moses MA, George AL, Sakakibara N, Mahmood K, Ponnamperuma RM, King KE, Weinberg WC. Molecular Mechanisms of p63-Mediated Squamous Cancer Pathogenesis. Int J Mol Sci. 2019 Jul 23;20(14):3590. doi: 10.3390/ijms20143590. PMID: 31340447; PMCID: PMC6678256.

5: Karrer S, Unger P, Spindler N, Szeimies RM, Bosserhoff AK, Berneburg M, Arndt S. Optimization of the Treatment of Squamous Cell Carcinoma Cells by Combining Photodynamic Therapy with Cold Atmospheric Plasma. Int J Mol Sci. 2024 Oct 8;25(19):10808. doi: 10.3390/ijms251910808. PMID: 39409136; PMCID: PMC11477452.

6: Farsam V, Basu A, Gatzka M, Treiber N, Schneider LA, Mulaw MA, Lucas T, Kochanek S, Dummer R, Levesque MP, Wlaschek M, Scharffetter-Kochanek K.

Senescent fibroblast-derived Chemerin promotes squamous cell carcinoma migration. Oncotarget. 2016 Dec 13;7(50):83554-83569. doi: 10.18632/oncotarget.13446. PMID: 27907906; PMCID: PMC5347788.

7: Bauwens E, Parée T, Meurant S, Bouriez I, Hannart C, Wéra AC, Khelfi A, Fattaccioli A, Burteau S, Demazy C, Fransolet M, De Schutter C, Martin N, Théry J, Decanter G, Penel N, Bury M, Pluquet O, Garmyn M, Debacq-Chainiaux F. Senescence Induced by UVB in Keratinocytes Impairs Amino Acids Balance. J Invest Dermatol. 2023 Apr;143(4):554-565.e9. doi: 10.1016/j.jid.2022.11.017. Epub 2022 Dec 15. PMID: 36528129.

8: Procopio MG, Laszlo C, Al Labban D, Kim DE, Bordignon P, Jo SH, Goruppi S, Menietti E, Ostano P, Ala U, Provero P, Hoetzenecker W, Neel V, Kilarski WW, Swartz MA, Brisken C, Lefort K, Dotto GP. Combined CSL and p53 downregulation promotes cancer-associated fibroblast activation. Nat Cell Biol. 2015 Sep;17(9):1193-204. doi: 10.1038/ncb3228. Epub 2015 Aug 24. Erratum in: Nat Cell Biol. 2015 Oct;17(10):1370. doi: 10.1038/ncb3242. PMID: 26302407; PMCID: PMC4699446.

9: Dotto GP. Calcineurin signaling as a negative determinant of keratinocyte cancer stem cell potential and carcinogenesis. Cancer Res. 2011 Mar 15;71(6):2029-33. doi: 10.1158/0008-5472.CAN-10-3750. PMID: 21406393; PMCID: PMC5539402.

10: Caini S, Raimondi S, Johansson H, De Giorgi V, Zanna I, Palli D, Gandini S. Telomere length and the risk of cutaneous melanoma and non-melanoma skin cancer: a review of the literature and meta-analysis. J Dermatol Sci. 2015 Dec;80(3):168-74. doi: 10.1016/j.jdermsci.2015.08.003. Epub 2015 Aug 22. PMID: 26341697.

11: Gache Y, Brellier F, Rouanet S, Al-Qaraghuli S, Goncalves-Maia M, Burty-Valin E, Barnay S, Scarzello S, Ruat M, Sevenet N, Avril MF, Magnaldo T. Basal Cell Carcinoma in Gorlin's Patients: a Matter of Fibroblasts-Led Protumoral Microenvironment? PLoS One. 2015 Dec 22;10(12):e0145369. doi: 10.1371/journal.pone.0145369. PMID: 26694869; PMCID: PMC4687848.

12: Nassir S, Yousif M, Li X, Severson KJ, Hughes A, Kechter J, Hwang A, Boudreaux B, Bhullar P, Zhang N, Butterfield RJ, Ma T, Leibovit-Reiben Z, Stockard A, Ogbaudu E, Costello CM, Nelson SA, DiCaudo DJ, Sekulic A, Baum CL, Pittelkow MR, Mangold AR. Multiomic Sequencing of Intermediate- to High-Risk Cutaneous Squamous Cell Carcinoma Identifies Critical Genes and Expression Patterns Associated with Disease and Poor Outcomes. J Invest Dermatol. 2025 Aug;145(8):2060-2070.e5. doi: 10.1016/j.jid.2025.01.015. Epub 2025 Jan 28. PMID: 39884456.

13: Azazmeh N, Assouline B, Winter E, Ruppo S, Nevo Y, Maly A, Meir K, Witkiewicz AK, Cohen J, Rizou SV, Pikarsky E, Luxenburg C, Gorgoulis VG, Ben-Porath I. Chronic expression of p16^{INK4a} in the epidermis induces Wnt-mediated hyperplasia and promotes tumor initiation. Nat Commun. 2020 Jun 1;11(1):2711. doi: 10.1038/s41467-020-16475-3. PMID: 32483135; PMCID: PMC7264228.

14: Morita N, Onodera S, Nakamura Y, Nakamura T, Takahashi SI, Nomura T, Azuma T. Keratinocytes from Gorlin Syndrome-induced pluripotent stem cells are resistant against UV radiation. Med Mol Morphol. 2021 Jun;54(2):69-78. doi: 10.1007/s00795-020-00264-4. Epub 2020 Aug 20. PMID: 32816116.

15: de Pedro I, Galán-Vidal J, Freije A, de Diego E, Gandarillas A. p21CIP1

controls the squamous differentiation response to replication stress. Oncogene. 2021 Jan;40(1):152-162. doi: 10.1038/s41388-020-01520-8. Epub 2020 Oct 23. PMID: 33097856.

16: Fujimura T, Asano Y. PAI-1 in Skin Malignancies: a Central Regulator of Tumor Progression and Therapeutic Resistance. Curr Treat Options Oncol. 2025 Nov;26(11):943-949. doi: 10.1007/s11864-025-01357-x. Epub 2025 Sep 26. PMID: 41003885; PMCID: PMC12552379.

17: Griewank KG, Murali R, Schilling B, Schimming T, Möller I, Moll I, Schwamborn M, Sucker A, Zimmer L, Schadendorf D, Hillen U. TERT promoter mutations are frequent in cutaneous basal cell carcinoma and squamous cell carcinoma. PLoS One. 2013 Nov 18;8(11):e80354. doi: 10.1371/journal.pone.0080354. PMID: 24260374; PMCID: PMC3832433.

18: Freyter BM, Abd Al-Razaq MA, Hecht M, Rübe C, Rübe CE. Studies on Human Cultured Fibroblasts and Cutaneous Squamous Cell Carcinomas Suggest That Overexpression of Histone Variant H2A.J Promotes Radioresistance and Oncogenic Transformation. Genes (Basel). 2024 Jun 27;15(7):851. doi: 10.3390/genes15070851. PMID: 39062630; PMCID: PMC11275829.

19: Toutfaire M, Dumortier E, Fattaccioli A, Van Steenbrugge M, Proby CM, Debacq-Chainiaux F. Unraveling the interplay between senescent dermal fibroblasts and cutaneous squamous cell carcinoma cell lines at different stages of tumorigenesis. Int J Biochem Cell Biol. 2018 May;98:113-126. doi: 10.1016/j.biocel.2018.03.005. Epub 2018 Mar 14. PMID: 29550586.

20: Li C, Sun C, Mahapatra KD, Riihilä P, Knuutila J, Nissinen L, Lapins J, Kähäri VM, Homey B, Sonkoly E, Pivarcsi A. Long noncoding RNA plasmacytoma

variant translocation 1 is overexpressed in cutaneous squamous cell carcinoma and exon 2 is critical for its oncogenicity. Br J Dermatol. 2024 Feb 16;190(3):415-426. doi: 10.1093/bjd/ljad419. Erratum in: Br J Dermatol. 2024 Jun 20;191(1):e1. doi: 10.1093/bjd/ljae157. PMID: 37930852.

21: Harada M, Jinnin M, Wang Z, Hirano A, Tomizawa Y, Kira T, Igata T, Masuguchi S, Fukushima S, Ihn H. The expression of miR-124 increases in aged skin to cause cell senescence and it decreases in squamous cell carcinoma. Biosci Trends. 2017 Jan 16;10(6):454-459. doi: 10.5582/bst.2016.01102. Epub 2016 Nov 5. PMID: 27818465.

22: Hida Y, Kubo Y, Arase S. Activation of fibroblast growth factor receptor 3 and oncogene-induced senescence in skin tumours. Br J Dermatol. 2009 Jun;160(6):1258-63. doi: 10.1111/j.1365-2133.2009.09068.x. Epub 2009 Mar 9. PMID: 19298285.

23: Natarajan E, Omobono JD 2nd, Jones JC, Rheinwald JG. Co-expression of p16INK4A and laminin 5 by keratinocytes: a wound-healing response coupling hypermotility with growth arrest that goes awry during epithelial neoplastic progression. J Investig Dermatol Symp Proc. 2005 Nov;10(2):72-85. doi: 10.1111/j.1087-0024.2005.200415.x. PMID: 16358814.

24: Ikeda H, Aida J, Hatamochi A, Hamasaki Y, Izumiyama-Shimomura N, Nakamura K, Ishikawa N, Poon SS, Fujiwara M, Tomita K, Hiraishi N, Kuroiwa M, Matsuura M, Sanada Y, Kawano Y, Arai T, Takubo K. Quantitative fluorescence in situ hybridization measurement of telomere length in skin with/without sun exposure or actinic keratosis. Hum Pathol. 2014 Mar;45(3):473-80. doi: 10.1016/j.humpath.2013.10.009. Epub 2013 Oct 19. PMID: 24411948.

25: Jin JY, Ke H, Hall RP, Zhang JY. c-Jun promotes whereas JunB inhibits epidermal neoplasia. J Invest Dermatol. 2011 May;131(5):1149-58. doi: 10.1038/jid.2011.1. Epub 2011 Feb 3. Erratum in: J Invest Dermatol. 2011 Jun;131(6):1388. PMID: 21289643; PMCID: PMC3108157.

26: Durinck S, Ho C, Wang NJ, Liao W, Jakkula LR, Collisson EA, Pons J, Chan SW, Lam ET, Chu C, Park K, Hong SW, Hur JS, Huh N, Neuhaus IM, Yu SS, Grekin RC, Mauro TM, Cleaver JE, Kwok PY, LeBoit PE, Getz G, Cibulskis K, Aster JC, Huang H, Purdom E, Li J, Bolund L, Arron ST, Gray JW, Spellman PT, Cho RJ. Temporal dissection of tumorigenesis in primary cancers. Cancer Discov. 2011 Jul;1(2):137-43. doi: 10.1158/2159-8290.CD-11-0028. Epub 2011 Jun 29. PMID: 21984974; PMCID: PMC3187561.

27: Choi SR, Chung BY, Kim SW, Kim CD, Yun WJ, Lee MW, Choi JH, Chang SE. Activation of autophagic pathways is related to growth inhibition and senescence in cutaneous squamous cell carcinoma. Exp Dermatol. 2014 Oct;23(10):718-24. doi: 10.1111/exd.12515. PMID: 25046976.

28: Li Y, Peart MJ, Prives C. Stxbp4 regulates DeltaNp63 stability by suppression of RACK1-dependent degradation. Mol Cell Biol. 2009 Jul;29(14):3953-63. doi: 10.1128/MCB.00449-09. Epub 2009 May 18. PMID: 19451233; PMCID: PMC2704755.

29: DeYoung MP, Johannessen CM, Leong CO, Faquin W, Rocco JW, Ellisen LW. Tumor-specific p73 up-regulation mediates p63 dependence in squamous cell carcinoma. Cancer Res. 2006 Oct 1;66(19):9362-8. doi: 10.1158/0008-5472.CAN-06-1619. PMID: 17018588.

30: Li X, Zhou Q, Wang S, Wang P, Li J, Xie Z, Liu C, Wen J, Wu X. Prolonged

treatment with Y-27632 promotes the senescence of primary human dermal fibroblasts by increasing the expression of IGFBP-5 and transforming them into a CAF-like phenotype. Aging (Albany NY). 2020 Aug 25;12(16):16621-16646. doi: 10.18632/aging.103910. Epub 2020 Aug 25. PMID: 32843583; PMCID: PMC7485707

31: Ke H, Harris R, Coloff JL, Jin JY, Leshin B, Miliani de Marval P, Tao S, Rathmell JC, Hall RP, Zhang JY. The c-Jun NH2-terminal kinase 2 plays a dominant role in human epidermal neoplasia. Cancer Res. 2010 Apr 15;70(8):3080-8. doi: 10.1158/0008-5472.CAN-09-2923. Epub 2010 Mar 30. PMID: 20354187; PMCID: PMC2855785.

32: Shin KH, Pucar A, Kim RH, Bae SD, Chen W, Kang MK, Park NH. Identification of senescence-inducing microRNAs in normal human keratinocytes. Int J Oncol. 2011 Nov;39(5):1205-11. doi: 10.3892/ijo.2011.1111. Epub 2011 Jul 1. PMID: 21725593; PMCID: PMC4004769.

33: Hori M, Suzuki K, Udono MU, Yamauchi M, Mine M, Watanabe M, Kondo S, Hozumi Y. Establishment of ponasterone A-inducible the wild-type p53 protein-expressing clones from HSC-1 cells, cell growth suppression by p53 expression and the suppression mechanism. Arch Dermatol Res. 2009 Oct;301(9):631-46. doi: 10.1007/s00403-008-0915-5. Epub 2008 Nov 14. PMID: 19009304.

34: Salmenperä P, Karhemo PR, Räsänen K, Laakkonen P, Vaheri A. Fibroblast spheroids as a model to study sustained fibroblast quiescence and their crosstalk with tumor cells. Exp Cell Res. 2016 Jul 1;345(1):17-24. doi: 10.1016/j.yexcr.2016.05.005. Epub 2016 May 10. PMID: 27177832.
